# Husbands

**Published:** 1891-08-01

**Authors:** 


### The Doctor's Armchair.

HUSBANDS.

"My intention is certainly to be a good woman," wrote Madame de la Charriere, "but there are a hundred thousand husbands with whom it would be extremely difficult to me, and with whom I should be sorry to answer for myself. God keep me from a fool!" From this it would seem that the lady, though such an exceedingly sensible person herself, thought it possible that she might one day come to marry a silly or a stupid man. There can be no doubt about it that sensible women do, not very infrequently, marry ill-conditioned or even wooden-headed men. The converse fact, that thinking men sometimes marry thoughtless and silly women, is also true. But that is relatively of less importance, because the husband, being the natural and proper head of the house, can do much to mitigate the effects of a wife's stupidity; while the wife, occupying only a secondary position, cannot so effectively counteract and repress a husband's folly. Speaking generally, it is much more necessary that a woman should have a sensible husband than that a man should have a rational wife.

"God keep me from a fool," was Madame Charriere's prayer. But what constitutes a fool from a wife's point of view? A man who is merely silly and inconsequent seldom succeeds in securing a wife for himself. The very fact that a male creature is married is some evidence that he has had a certain amount of critical faculty in that he has made a choice; and also a certain amount of will and resolution in that he has been able to "screw his courage to the sticking place" to ask the question of questions. Husbands, however, who know for various reasons that they are not fools of the silly and inconsequent type, must not, therefore, conclude that they are not fools in any sense at all. What do their wives think of them? Women, it may be taken for granted, do not look at men from the standpoint of the man, but from that of the woman. When Madame Charriere besought heaven not to give her a fool for a husband, it is pretty certain that she had in her own mind a very clear idea of what she meant by a fool.

The average husband will admit that it is very desirable for him to have his wife's good opinion. That he cannot have without deserving it, at least to a certain extent. A good wife can invent many excuses for her husband; and it is wonderful how long she can continue to satisfy her own mind with them. But there is a limit to such excuse-making and satisfaction; and when once a wife has made up her mind against her husband, a very desperate point has been reached in that particular domestic history. Women, it is often said, do not reason. But though they may not reason, according to Whately, they undoubtedly think, and think both positively and obstinately. Madame Charriere evidently felt that if she should be so unlucky as to have a fool for her husband, she did not know what desperate consequences might not follow. She might go so far as to leave him; in the last tremendous resort she might even be tempted to shoot or poison him.

But Madame Charriere was a woman of the last century, and she lived, therefore, at a period when there was much more inequality of intellectual position between husband and wife than there is now. The average woman has advanced amazingly both in intellectual culture and in the love of freedom and equality during the present century. She is a much keener critic than her grandmother was; and it is probable that her patience has diminished as her critical power has increased. The husband who is in any sense a fool in these times must expect that his wife will know exactly how he should be labelled, and will not hesitate, at any rate in her own mind, to pin on the label in its proper place.

Let us now return to our former question, and ask what it is which constitutes a husband a fool in the eyes of a patient and affectionate wife? Probably what the average woman feels most acutely in her husband's behaviour is his want of justice and chivalry towards herself. A man may have a perfectly clear head for business, and may conduct both his public and social affairs with excellent success, and yet in the inner privacy of his home he may be egotistical, exacting, unjust, and generally unbearable. He may be all these without intending it. Many people who recognise the duty of behaving in a just and manly way outside seem to think that the home is a place where all restraint is to be thrown off, and where unalloyed selfishness is to dictate every act and word. There are men who look upon wives, children, servants, friends, and home as made for their own exclusive pleasure and advantage in every way. Duty, honour, and general good behaviour may be characteristic of them in business and in outside social affairs, but at home quite different feelings and habits usurp the place of duty and good behaviour and make a man who is esteemed by the world hated, feared, and despised by his wife. Such a man is a fool. It was probably some such man that Madame Charriere had in her mind when she implored Providence to save her from having a fool for a husband.

The average man is seen either at his best at home, or at his worst. If he is a noble man down to the very roots of his nature, the home, which sees more of him than any other place, will know his value best. But all men are not noble down to the very roots of their nature. The different ingredients of the average man's character almost appear as if they had been put in by different hands. The same man may seem, and even be, generous at one time and selfish at another; brave under one set of circumstances and cowardly under a different set; patient and forbearing to-day; irritable, irrational, and unjust to-morrow. The wife perceives all these diversities of feeling and conduct. In some women the critical eye is keenest, in others the eye of a tender affection. But affection may be more painfully conscious of faults in a beloved person than even adverse criticism. When a husband is a fool his wife may know it and pity him, or she may know it and hate him. It is better to be pitied by your wife than hated; but it is best of all to be trusted.

The part of husband is difficult to play if it is to be played well. A man should always and everywhere be at his best; at home he should be better than his best. It is at home or at prayer that a man is in the most favourable circumstances for being at his best. In neither of these circumstances is there any temptation to act a part, to play the hypocrite. Almighty God takes the justest and most charitable view of a man which can be taken. A good wife takes the most charitable, and does her best to take the most just. On the whole, one is inclined to say that if a man be not a good husband, he can hardly be good at all. The home is the most delicate and sensitive of all testing places; and it is probable that he who fails there will at the greatest of all judgments finally prove a failure.

